# Effects of Occupational Fatigue on Cognitive Performance of Staff From a Train Operating Company: A Field Study

**DOI:** 10.3389/fpsyg.2020.558520

**Published:** 2020-09-11

**Authors:** Jialin Fan, Andrew P. Smith

**Affiliations:** ^1^School of Psychology, Shenzhen University, Shenzhen, China; ^2^Centre for Occupational and Health Psychology, School of Psychology, Cardiff University, Cardiff, United Kingdom

**Keywords:** occupational fatigue, rail industry, rail staff, field study, performance

## Abstract

**Background:**

Occupational fatigue is a key issue in the rail industry that can endanger staff, passenger, and train safety. There is a need to demonstrate the relationship between workload, fatigue, and performance among rail staff.

**Objective:**

The present study, conducted in the workplace in realistic situations, integrating both subjective and objective measurements, aimed at demonstrating the relationship between workload, fatigue, and cognitive performance with a rail staff sample.

**Methods:**

The “After-Effect” technique was applied in the current study. Online diaries and cognitive performance tasks were used to assess the fatigue, work experiences, and performance of rail staff before and after work on the first and last days of one working week.

**Results:**

Reported fatigue was greater after work on both the first and last day of the working week. There were large individual differences in the change in fatigue and workload ratings. Analysis of covariance with age and the pre-work performance score as covariates and the post-work performance score as the dependent variable showed that high levels of fatigue were associated with impaired performance on both the visual search and logical reasoning tasks. Workload had fewer effects on performance than fatigue.

**Conclusion:**

This field study provided evidence for the relationship between work-related fatigue and performance impairment. The findings show the need for future work on predicting fatigue-related performance decrements, and the necessity of providing interventions and support so that the risk to safety can be reduced.

## Introduction

Fatigue is often an indicator of an unhealthy lifestyle. It has found to be associated with higher probability of illness and injury in the workplace (Harma et al., 1998, 2002; Chau et al., 2008). Fatigue is synonymous with a generalized stress response over time. Occupational fatigue may occur during or after work; it may also occur before work when the worker has not fully recovered from previous fatigue through the regular periods of rest before the onset of the next set of demands (Cameron, 1973). It has been found to be associated with impaired cognitive performance, including increased reaction time, decreased vigilance, perceptual and cognitive distortions (reviewed in Krueger, 1989), dropped skill effectiveness (Drew, 1940). Fatigue also leads to impaired memory and information processing (Craig and Cooper, 1992), reductions in concentration, motivation, and activity (Beurskens et al., 2000). In addition, fatigue may impair the sense of agency (i.e., the loss of the sense of being responsible for own’s actions; Howard et al., 2016), which increases the safety risks in the workplace. In particular, previous studies show that human agency was reduced by increased out of the loop events which could be associated with fatigue, and decreased control or increased automation in the environment (Berberian et al., 2012; Kumar and Srinivasan, 2012, 2013; Moore, 2016; Di Plinio et al., 2019, 2020). Such effects may also show inter-individual differences (Di Plinio et al., 2019) and also vary with cultural background (Barlas and Obhi, 2014).

In the railway industry, occupational fatigue is a severe problem which jeopardizes not only the staff health but also train and passenger safety, as most jobs are safety-critical. Evidence for fatigue among rail staff has been found in previous studies, in which various methods have been used, including surveys (Cotrim et al., 2017; Fan and Smith, 2017), incident reports (reviewed in Buck and Lamonde, 1993; Ugajin, 1999; Fan and Smith, 2018), simulated driving studies (Dorrian et al., 2007), and interviews (Filtness and Naweed, 2017). In particular, fatigue is considered to be a causal or contributory factor in the majority of train accident and incident investigation reports (British Rail Safety and Standards Board, 2005; British Rail Accident Investigation Branch, 2008, 2010). Fatigue, its impact on task performance, and fatigue-related human errors have been found in previous research in several different transport sectors (e.g., road drivers: Feyer and Williamson, 2001; seafarers: Smith et al., 2006), and has also been suggested as a key issue for train safety (Bowler and Gibbon, 2015). However, the field of rail fatigue research was historically smaller than that of other transport sectors, and the investigation of the effect of fatigue on performance in real time in the workplace is still lacking in this industry.

The causes of occupational fatigue can emanate from either inside or outside the workplace, and mainly include task-related factors and sleep-related factors. Jobs in the rail industry were designed to operate on a 24/7 basis, often with an irregular schedule. A large-scale study (Fan and Smith, 2017) identified the main predictors of fatigue in the rail industry as high job demands (i.e., workload), shift-work, poor job control and support, and noise and vibration in the working environment. Shift work, especially the night and early morning shifts, disrupts the sleep–wake cycle (Ferguson et al., 2008) and deprives workers of sleep (Åkerstedt, 1991). Shift workers have little time to recover when working certain shift hours, which makes them more likely to suffer from cumulative fatigue (Åhsberg et al., 2000). Moreover, Dorrian et al. (2011) indicated that in addition to work hours and sleep length, workload significantly influenced fatigue among train crew. It is notable that mental workload is the major problem in the modern railway industry rather than traditional physical workload, due to the increasing level of automation in operating systems (Young et al., 2015; Fan and Smith, 2019). The majority of job tasks in this modern industry require more cognitive demands (e.g., selective attention, sustained vigilance), resulting in a heavy mental workload and increased fatigue; meanwhile, fatigue is associated with a deterioration of attention and impaired performance. Failure to maintain such performance at an acceptable level brings danger, especially to those working in safety-critical job roles.

Subjective measurement of fatigue has been validated as a reliable way to distinguish between fatigued and non-fatigued staff (e.g., Chalder et al., 1993; Kim et al., 2010), and this is widely used in different types of job disciplines, both within (Kishida, 1991) and between industries (Kogi et al., 1970; Beurskens et al., 2000). Recently, however, Cheng and Hui-Ning (2019) argued that the ability of rail staff to perceive their own fatigue could be limited, which may due to sleep debt and cumulative sleep loss, particularly following a string of atypical shifts (night or early morning shift). Therefore, it is important to also include objective measurement of fatigue and performance which can be used in the work situation along with subjective measurement to reducing potential subjective biases. However, it can be a challenge to apply certain objective measurements in the railway environment. Railway companies usually have their own rules regarding staff uniforms for consistency and safety, which means that wearing extra instruments for objective measurement, such as Electroencephalography (EEG) or eye-tracking equipment, is not allowed in the workplace, as it may cause distractions and other potential safety risks.

Broadbent (1979) suggested that using the “After-Effect” technique in fatigue measurement could be applicable in realistic situations. This involves measuring performance before and after a specific task or work period, without changing people’s normal behaviors during and after the task. The after-effect symptoms of fatigue usually include longer reaction times and reduced accuracy. In the work context, the After-Effect method compares the difference in performance before and after work, and a greater difference reflects a greater effect. This method has already been widely used in workload studies (e.g., Parkes, 1995; Hockey and Earle, 2006). For instance, workload study Parkes’s (1995) found that reaction time and accuracy in search tasks and logical reasoning ability showed clear impairments due to the effect of higher workload. It has also been used to assess other factors which contribute to fatigue, such as the common cold (Smith et al., 2000), caffeine (Brice and Smith, 2001; Smith, 2002; Doherty and Smith, 2005), and night work (Åkerstedt, 1988). Recently, Smith and Smith (2017) used the After-Effect method to assess rail engineers’ fatigue and performance on the first and last day of the work week and showed that the extent of fatigue could be identified using this methodology.

Online fatigue measures could be a more appropriate tool for detecting fatigue in the workplace due to their convenience and low development cost. Online cognitive tests have been used for the past two decades, and a review of them confirmed their ability to provide realistic simulations of cognitive tasks in daily life, which is the main advantage of computerized cognitive evaluation (see Crook et al., 2009). It is possible for online measures to be used in the workplace and they are often more convenient than offline tests or the use of measures from laboratory experiments. One fatigue study with students, which used a methodology that combined the After-Effect method and online cognitive performance tasks to measure fatigue in a real-life setting (Fan and Smith, 2017), established the relationship between workload, fatigue, and cognitive performance. This study showed that workload increased subjective fatigue after work which then resulted in cognitive performance impairments, including slower reaction time and decreased accuracy, while the effect of time of day on performance was not found significant. However, this study consisted of undergraduate students with risk factors for fatigue due to their study life at university, which are different from fatigue in the actual work life of the railway industry. Thus, a further experiment based on a staff sample is needed.

The present study aimed to use this same methodology to demonstrate the relationship between workload, fatigue, and objective performance with staff from a train operating company. The company was interested in generic fatigue across a range of jobs. Other research has adopted the present approach to study train drivers (Evans, 2019), conductors, guards, and engineers (Smith and Smith, 2017). The methods used in this study consisted of a self-assessment diary, mainly used to record ratings of fatigue and workload, and also objective performance tests. The experimental hypothesis for this study predicted that an increased feeling of fatigue would lead to performance reduction, including delayed reaction time, and lower accuracy rates in both visual search and logical reasoning tests. This methodology was also used to examine whether the effects of fatigue and workload were different.

## Materials and Methods

### Participants

This study recruited participants with different types of jobs from volunteers from a train company in the *United Kingdom* [*N* = 19, mean (± SD) age = 41.86 ± 9.89 yrs.; 74% male], as all job types may be susceptible to fatigue (Fan and Smith, 2017). The main job types reported were managers, conductors, drivers, station workers, engineers, and administrators. Selection of different job types meant that any obtained results could be generali***z***ed across occupations. Participants were fit for work but no other data was collected on health status.

### Procedure

This study included four sessions in total, requiring participants to complete the diary and the tests immediately before starting work, and immediately after finishing work on the first and fourth days of a working week. For example, if one participant was off-work on Tuesday and Wednesday, and then worked the following four continuous days, this participants would complete the diary on Thursday (the first day of his or her working week) and on Sunday (the last day of his or her working week). An invitation e-mail with attached information about the study and an informed consent form was sent to potential participants. Once participants had signed and returned the forms, they were asked to provide the start date of their next work week with four continuous days of working. The links to the four test sections and a familiarization session were then sent to them. The familiarization session included an introduction to the diary and an example of each cognitive task to ensure that the participants were able to complete the tasks correctly before starting the study. On the testing day(s), participants were asked to complete the online diary and cognitive tasks immediately before starting work and immediately after finishing work via a computer or mobile phone.

Participants were free to withdraw from the survey at any point. This study was reviewed and approved by the School of Psychology Research Ethics Committee at Cardiff University and carried out with the informed consent of the volunteers.

### Materials

The materials used in this study included a diary and two online cognitive tasks and took about 15 min to complete. These online measures required assessment by mobile phone or computer, and participants responded by touching the screen (if using mobile phone) or clicking on the mouse (if using the computer). All the tasks and data collection were via the Qualtrics online survey platform.

#### Diary

The diary was used to measure fatigue and the causes of fatigue. It consisted of 15 single-item questions, including six questions to be answered before work and nine questions to be answered after work. Supplementary Table 1 (in Supplementary Material) shows the details of the diary questionnaire. It was designed based on the material used in Smith and Smith’s (2017) diary studies, and majority questions were on a 10-point scale. The questions in the pre-work diary covered sleep duration and quality, commute time, fatigue due to the commute, general health status, and alertness before starting work. The questions in the post-work diary recorded workload, effort, fatigue, stress, break duration, work duration, time of work completion, and level of distraction during work. There were extra questions in the post-work diary on the last day, which asked whether the participants had worked at the same time every workday during the working week; if participants answered no, they were asked about their working time for each day.

#### Online Cognitive Tasks

Two online cognitive tasks were used to assess objective performance in each session: a visual search and a logical reasoning task. These two tests have been widely used in previous workload (e.g., Parkes, 1995) and fatigue studies (e.g., Lamond and Dawson, 1999; Barker and Nussbaum, 2011). The online version of such tests was validated in our previous study with the student sample (Fan and Smith, 2017; Fan, 2019). Supplementary Figures 1, 2 (in Supplementary Material) shows the example of a trial of each task. For both tasks, the inter-trial intervals were 500 ms. The tasks were distributed and the data collection was completed via the Qualtrics online survey platform. Participant would assess the task using either computer or mobile phone, responding by clicking mouse or tapping touch screen.

The visual search task consisted of 12 trials, which randomly appeared from a total of 30 possible trials. In each trial, participants were randomly shown a 60-letter set and one target letter. They were required to find and click the target letter as quickly and accurately as possible on the screen. The response time and accuracy for each trial were recorded.

The logical reasoning task consisted of 24 trials and required the participants to make a decision between two options as quickly and accurately as possible. This test was based on Baddeley’s (1968) grammatical reasoning test. The outcome measures were response time and accuracy.

## Analysis

Both the diary and the cognitive tasks were presented online using the Qualtrics software package. The diary and performance data were then downloaded into a single SPSS data file. Analysis was carried out using the IBM SPSS 25 package. The main focus of the analysis was on the associations between fatigue and workload and changes in performance over the day. Analyses of covariance with the pre-work measures and age as covariates, and the post-work performance scores as dependent variables were carried out. Fatigue and workload change scores were split into high and low groups (based on the median of scores from these questions in the diary) and these were the between subject factors in the analyses of covariance. Fan and Smith (2017), in a study of university students, found that fatigue reduced visual search accuracy and led to slower logical reasoning speed. Workload had no significant effects. One-tail significance levels were used where the two tailed level was not significant, as it was predicted that high fatigue and high workload would be associated with impaired performance seen in the Fan and Smith (2017) study.

## Results

### The Sample

Nineteen participants, 14 of whom were male, completed the whole study. The most common job types reported were managers (26.3%), engineers (15.8%), conductors (15.8%), drivers (15.8%), and station workers (15.8%), followed by administrators (10.5%). Most participants did daytime shifts (68.4%), while 31.6% did night shifts (begin between the hours of 7:00 p.m. and 12:00 a.m.) or early morning shifts (begin between the hours of 12:00 a.m. and 6:00 a.m.). Nearly half (43.1%) of the participants worked two or more different shift times during the testing week (4 days).

### Fatigue and Workload Ratings

The descriptive statistics for the fatigue ratings and the performance tasks, are shown for pre-work and post work on the first and last day of the working week in Table 1.

**TABLE 1:** Descriptive statistics for fatigue ratings and performance tests (mean [SD]).

	Day 1 Pre-work	Day 1 Post-work	Day 4 Pre-work	Day 4 Post-work
Fatigue ratings (scale of 1–10; high scores = greater fatigue)	2.16 [1.21]	6.42 [2.12]	2.47 [1.61]	7.11 [2.00]
Visual search accuracy (% correct)	97.81 [4.68]	97.81 [4.68]	94.07 [5.77]	90.35 [8.45]
Visual search speed (s)	13.58 [3.15]	14.25 [3.00]	13.93 [3.54]	14.29 [3.06]
Logical reasoning accuracy (% correct)	74.34 [20.00]	77.63 [21.35]	74.78 [22.27]	79.39 [23.63]
Logical reasoning speed (s)	6.28 [1.73]	6.94 [2.39]	5.58 [1.40]	5.66 [1.38]

On the first working day, fatigue ratings showed a large increase over the day (pre-work mean = 2.16; post-work mean = 6.42). There was considerable variation across individuals with the increase in fatigue having a range from 0 to 800%. A similar profile was seen for the last working day (pre-work mean: 2.47; post-work mean = 7.11), and again there were large individual differences in the change of fatigue over the day (range = −14–900%). Workload ratings were consistent across days (Day 1: mean = 5.79, SD = 2.18; Day 4: mean = 5.42, SD = 2.43) and showed large individual differences (Day 1: range = 1–9; Day 4: range = 1–9).

Changes in fatigue over the day were correlated with age (Day 1: *r* = 0.73, *p* < 0.001). On the last day increased fatigue was associated with greater distraction due to thinking about other things (*r* = 0.49, *p* < 0.05). Workload ratings were associated with ratings of effort (*r* = 0.61, *p* < 0.01), stress (*r* = 0.49, *p* < 0.05), and alertness (*r* = −0.49). These results show that fatigue and workload are different constructs which are only weakly correlated.

### Changes in Fatigue, Workload and Performance Changes Over the Day

Analyses of covariance were carried out to examine associations between changes in fatigue, workload and performance after work. Changes of fatigue and workload were divided into high and low groups using a median split. Before work performance measures were covariates for the corresponding after work measure (the dependent variable). Age was also included as a covariate. It was predicted that increases of fatigue and workload would be associated with impaired performance.

**FIGURE 1:**
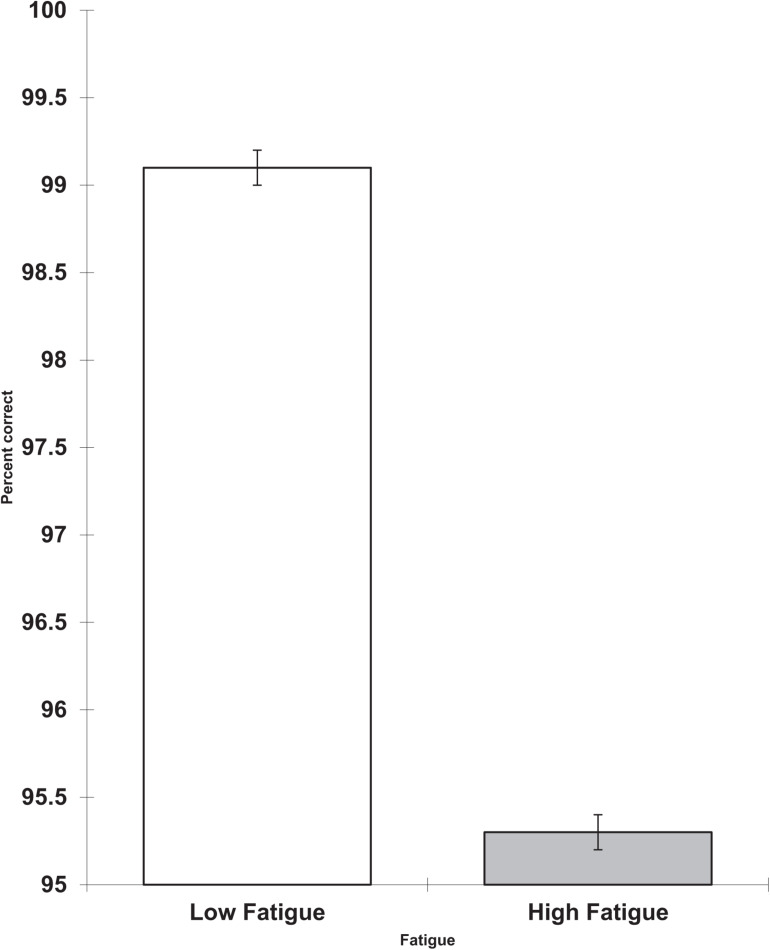
Change in fatigue on Day 1 and visual search percent correct (scores are the adjusted means, standard errors (s.e.s) shown as bars.

On the first day, the high fatigue group had less accurate performance on the visual search task than the low fatigue group (*F* = 3.78, df = 1.13, *p* = 0.037, 1-tail, partial eta squared = 0.225). This result is shown in Figure 1.

**FIGURE 2:**
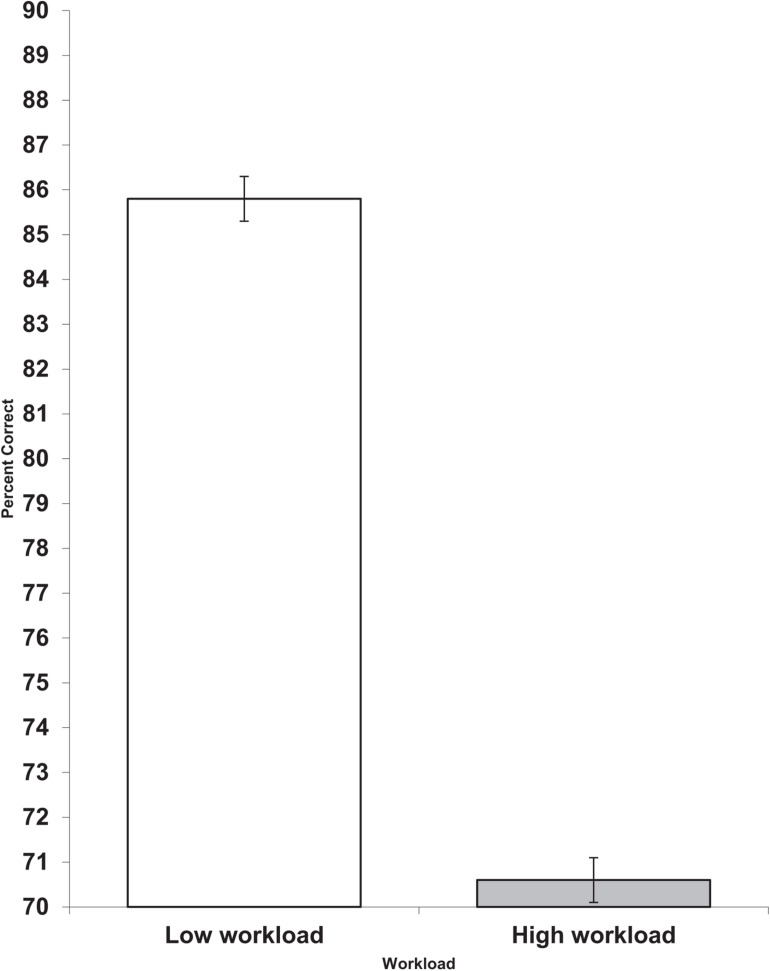
Day 1 workload and logical reasoning percent correct (scores are the adjusted means, s.e.s shown as bars).

Also on Day 1, the high workload group had less accurate performance on the logical reasoning task than the low workload group (*F* = 5.37, df = 1,13, *p* = 0.037, partial eta squared = 0.292). This result is shown in Figure 2. None of the other effects were significant.

**FIGURE 3:**
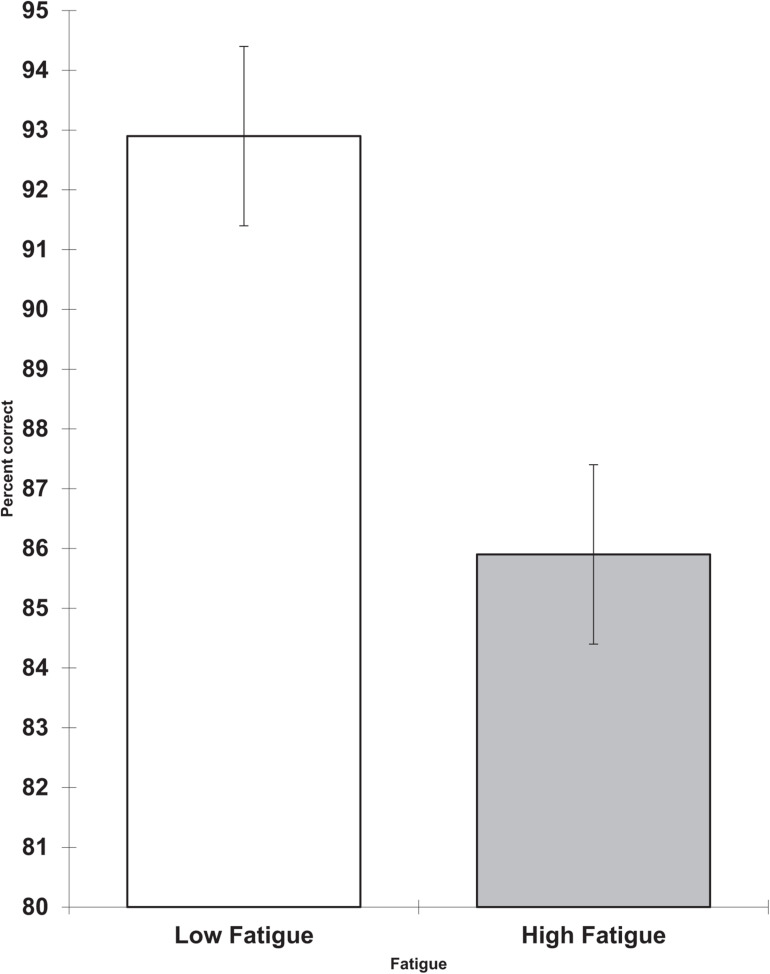
Day 4 fatigue and visual search percent correct (scores are the adjusted means, s.e.s shown as bars).

On the last working day, the high fatigue group again had less accurate performance on the visual search task (*F* = 5.84, df = 1,13, *p* = -0.031, partial eta squared = 0.310). This is shown in Figure 3.

**FIGURE 4:**
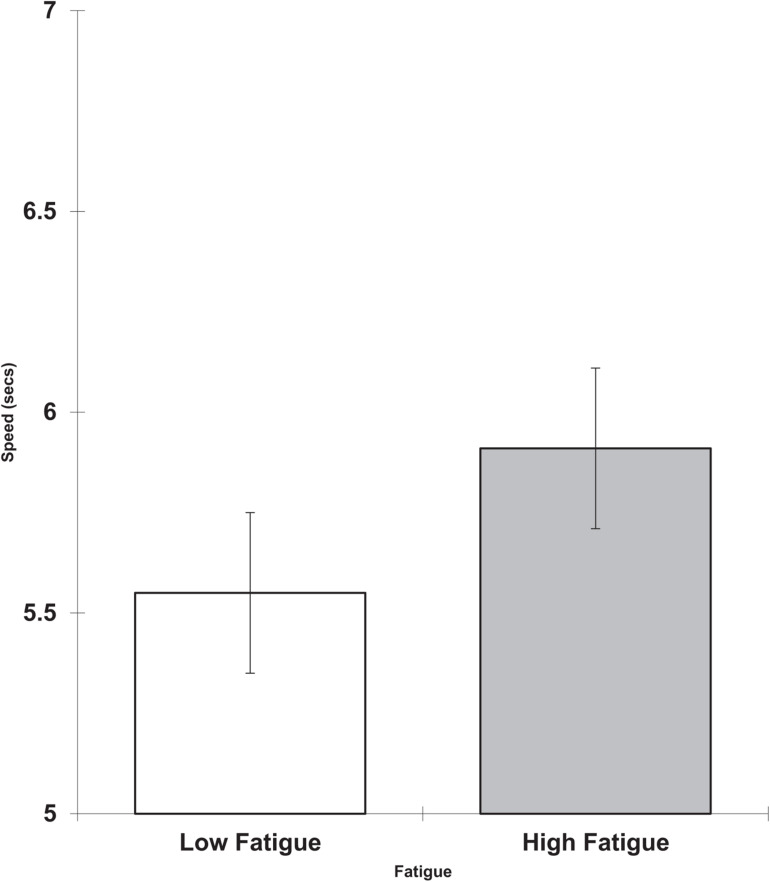
Day 4 fatigue and logical reasoning speed (seconds per item; scores are the adjusted means, s.e.s shown as bars).

The high fatigue group were also slower than the low fatigue group on the logical reasoning task on the last day (*F* = 3.38, df = 1,13, *p* < 0.045, 1-tail, partial eta squared = 0.206). This is shown in Figure 4.

## Discussion

The present research involved a field study using online fatigue tests integrating both subjective and objective measurements, which was validated in a previous fatigue study (Fan and Smith, 2017). The design of the fatigue tests combined online methods and the After-Effect technique. This methodology was suitable and convenient to use in the workplace, especially in the railway industry where wearing extra instruments of objective measurement was not allowed, as this might create distractions and pose other potential safety risks.

Overall, the results of this study with the staff sample were in line with those of previous studies, including our study with a student sample using the same performance tasks (Fan and Smith, 2017) and those carried out in different transport industries (Feyer and Williamson, 2001; Smith et al., 2006; Smith and Smith, 2017) which found that performance was impaired by fatigue. The effects of fatigue on cognitive performance were found with both high and low workloads. However, there was some evidence of independent effects of workload on performance speed, although such effects were less frequent than those of fatigue. In addition, subjective fatigue increased, and general outcomes got worse at the end of the week, suggesting an effect of cumulative work fatigue on outcomes throughout the working week. This result was very similar to fatigue observed in seafarers, which increased day by day during the tour of duty and continued into leave (Bal et al., 2015).

The main hypothesis of the current study predicted that increased occupational fatigue would lead to performance reduction, including slower RT and lower accuracy rates. Comparable to our student sample study (Fan and Smith, 2017), the results here showed that an increased feeling of fatigue was associated with impaired performance, including decreased accuracy in the visual search task and slower RT in the logical reasoning task, which supports this hypothesis. The effects of workload were restricted to less accurate performance of the logical reasoning task on the first day.

This study was performed in the United Kingdom and the results were obtained using a United Kingdom sample, and differences in culture (Barlas and Obhi, 2014) were not relevant here. Previous studies have shown that fatigue impaired cognitive performance (e.g., Craig and Cooper, 1992; Beurskens et al., 2000), which was supported by the current study. The results from previous studies also suggest that it could be the increased fatigue, decreased control, and increased automation in the working environment which resulted in the changed sense of agency (e.g., Berberian et al., 2012; Kumar and Srinivasan, 2012, 2013; Moore, 2016; Howard et al., 2016; Di Plinio et al., 2019, 2020). The modern railway industry has increased the level of automation in operating systems and decreased control by operators (Young et al., 2015; Fan and Smith, 2019), and future studies of fatigue in railway staff samples should focus on changes in the sense of agency.

This study investigated the effect of occupational fatigue on cognitive performance in railway staff, and its results provide insight on current practices regarding fatigue management in the industry. The findings allow us to offer a few suggestions for the railway industry. In general, either organizations or individuals should raise the issue of fatigue and its after-effects, and take action to prevent and manage it and related impaired performance in the workplace. The present research, in line with previous studies (e.g., Dorrian et al., 2011; Fan and Smith, 2017), indicated that workload should be considered as well as fatigue. Considering the nature of jobs in the railway industry, however, it will be not easy to control or reduce the workload, especially with the unpredictability of train problems and unplanned overtime work. Thus, companies and organizations can apply such online fatigue self-assessment and cognitive performance tasks to assess staff fatigue level before and after work. For those with no indication of fatigue, this will not change the normal working behavior of during their duty. For those with fatigue this can be prevented and managed by providing support (e.g., fatigue managing advices or intervention) during times when fatigue is likely to be at a high-risk level. Also, companies may need to improve work patterns and arrange rest times during and after work for recovery from fatigue, which reduces the risk of future fatigue-related performance impairment.

### Limitations

The first sets of limitations are common in diary studies. As a method, the online diary study is less controlled than laboratory experiments, although it has the advantage of assessing the effects of fatigue in the context of participants’ daily work lives, as well as being able to assess the effect of cumulative fatigue for a longer period of time than in laboratory experiments. One issue is completion of the diary at the correct time. One participant commented that he did not have time to complete the diary immediately after work because he was off very late and caught transport to return home in a hurry. Although this participant completed the post-work diary immediately upon arriving home, his fatigue and performance may have recovered during the commute. Another problem is the completion of the study. Diary studies are also time-consuming, and participants required reminders and encouragement to fully complete the diaries. In this study, it was difficult to recruit participants and have them fully complete all of the four sessions, especially the post-work diary on the last day of the work week. The majority of participants who forgot to fill in the last diary decided to quit the study rather than re-do it. This meant that the major limitation of the present study was the small sample size. The small sample size also meant that it was not possible to consider individual differences, such as job type or the personality of the participants.

### Future Research

The current study was an initial trial of studying the effect of fatigue on performance in a real-life setting. There is a plan to conduct more staff experiments to further investigate the effects of fatigue on performance, as well as intervention experiments. Future research requires better control of online diary data collection. While the online diary is an advanced method for assessing fatigue closely in the context of daily work life, reminder texts or e-mails are needed to ensure that participants fill out each diary on time. The diary could be integrated with the HSE Fatigue and Risk Index (a fatigue prediction tool based on shift patterns currently used in the United Kingdom rail industry) in a future study. Although the job demands variable in this index is usually set at a constant level for all staff, it can be measured through the single-item self-assessment in the diary.

## Conclusion

Occupational fatigue is an important issue in the rail industry and it can endanger passenger, staff and train safety. It is also important in jobs which are not safety critical as it can influence the efficiency of the organization and the health and wellbeing of staff. Our previous research has examined this issue in drivers (Evans, 2019), conductors, guards, and engineers (Smith and Smith, 2017). There is now a need to demonstrate the relationship between workload, fatigue, and performance among a wider range of staff of train operating companies. The present study was carried out in the workplace using an online methodology with both subjective and objective measurements. The aim was to examine the relationship between workload, fatigue, and cognitive performance using staff from a train operating company. The “After-Effect” technique was used with online diaries and cognitive performance tasks assessing the fatigue, work experiences, and performance of staff before and after work on the first and fourth days of one working week.

This field study provided evidence for the relationship between work-related fatigue and performance impairment. The findings show the need for future work on predicting fatigue-related performance decrements, and the necessity of providing interventions and support so that the risk to safety can be reduced. The results demonstrated that the objective performance of staff was impaired due to fatigue, shown as decreased accuracy on a visual search task and the logical reasoning task. These findings were in line with those of previous research in other work contexts. Increased fatigue was associated with higher workload, while fatigue before work was also associated with the quality and duration of sleep. Considering it is not easy to control or reduce the workload due to the nature of the jobs, the rail industry could focus instead on improving the guidelines regarding rest to manage fatigue, which would then reduce the risk of work performance impairment. Future research using an online diary should consider recruiting a larger sample and mitigating the risk of absent or incomplete diary entries.

## Data Availability Statement

The raw data supporting the conclusions of this article will be made available by the authors without undue reservation.

## Ethics Statement

The studies involving human participants were reviewed and approved by School of Psychology Research Ethics Committee at Cardiff University. The patients/participants provided their written informed consent to participate in this study.

## Author Contributions

AS formulated the research question, designed the study, and revised the manuscript for important intellectual content. JF conducted the analyses, interpreted the data, and drafted the original manuscript. Both the authors approved the final version for publication and also agreed to be held accountable for all aspects of the work in ensuring that questions related to accuracy and integrity are appropriately investigated and resolved.

## Conflict of Interest

The authors declare that the research was conducted in the absence of any commercial or financial relationships that could be construed as a potential conflict of interest.
