# Supplementary material for: Effects of Occupational Fatigue on Cognitive Performance of Staff From a Train Operating Company: A Field Study
Source: Front Psychol. 2020 Sep 11;11:558520. doi: 10.3389/fpsyg.2020.558520 (PMC7517727; doi:10.3389/fpsyg.2020.558520)

Supplementary Material

Systematic Review of Fatigue among Rail Staff

Jialin Fan, Andrew P. Smith^*^

*** Correspondence:** Andrew P. Smith: [SmithAP@Cardiff.ac.uk](mailto:SmithAP@Cardiff.ac.uk)

# Supplementary Tables

**Supplementary Table 1.** Questiona in the Diary

| **Before Work Diary** |
| --- |
| 1. How many hours sleep did you get last night?  This question asks about your recent sleep experience, no matter it was at daytime or at night.  ________ hours ________ minutes |
| 2. How was the quality of your sleep?  Not at all good Very good  1 2 3 4 5 6 7 8 9 10 |
| 3. How long did it take you to travel to work?  ________ hours ________ minutes |
| 4. How fatigued did you feel from your commute?  Not at all Very fatigue  1 2 3 4 5 6 7 8 9 10 |
| 5. How well are you feeling now?  Not at all well Very well  1 2 3 4 5 6 7 8 9 10 |
| 6. How alert do you feel now?  Not at all Very alert  1 2 3 4 5 6 7 8 9 10 |

| **After Work Diary** |
| --- |
| 1. How was your workload today?  Very low Very high  1 2 3 4 5 6 7 8 9 10 |
| 2. How much effort did you have to put into your job today?  Very little A great deal  1 2 3 4 5 6 7 8 9 10 |
| 3. How fatigued do you feel now?  Not at all Very fatigue  1 2 3 4 5 6 7 8 9 10 |
| 4. How stressed do you feel now?  Not at all Very stressed  1 2 3 4 5 6 7 8 9 10 |
| 5. What was the total length of your breaks today?  ________ hours ________ minutes |
| 6. What was the total length of your work today?  ________ hours ________ minutes |
| 6.1. What time did you start work today? (e.g. Hour: 23 Minute: 30)  ________ hours ________ minutes |
| 6.2. What time did you finish work today?  ________ hours ________ minutes |
| 7. During your work today, to what extent were you thinking about other things rather than work?  Not at all Very much so  1 2 3 4 5 6 7 8 9 10 |
| 8*. Did you work at the same time on other days of this week? (start time, end time, and length)  Yes No  8.1* If no, which day(s) did you work at a different time? And what was the total length of your work on that day(s)? (hours, minutes)  _________________________________ |
| 8.2* What time did you start and finish work on each of those days? For example, Day 2 - 6.30 am  _________________________________ |

* The question only asked in the after-work diary on the last day.

# Supplementary Figure


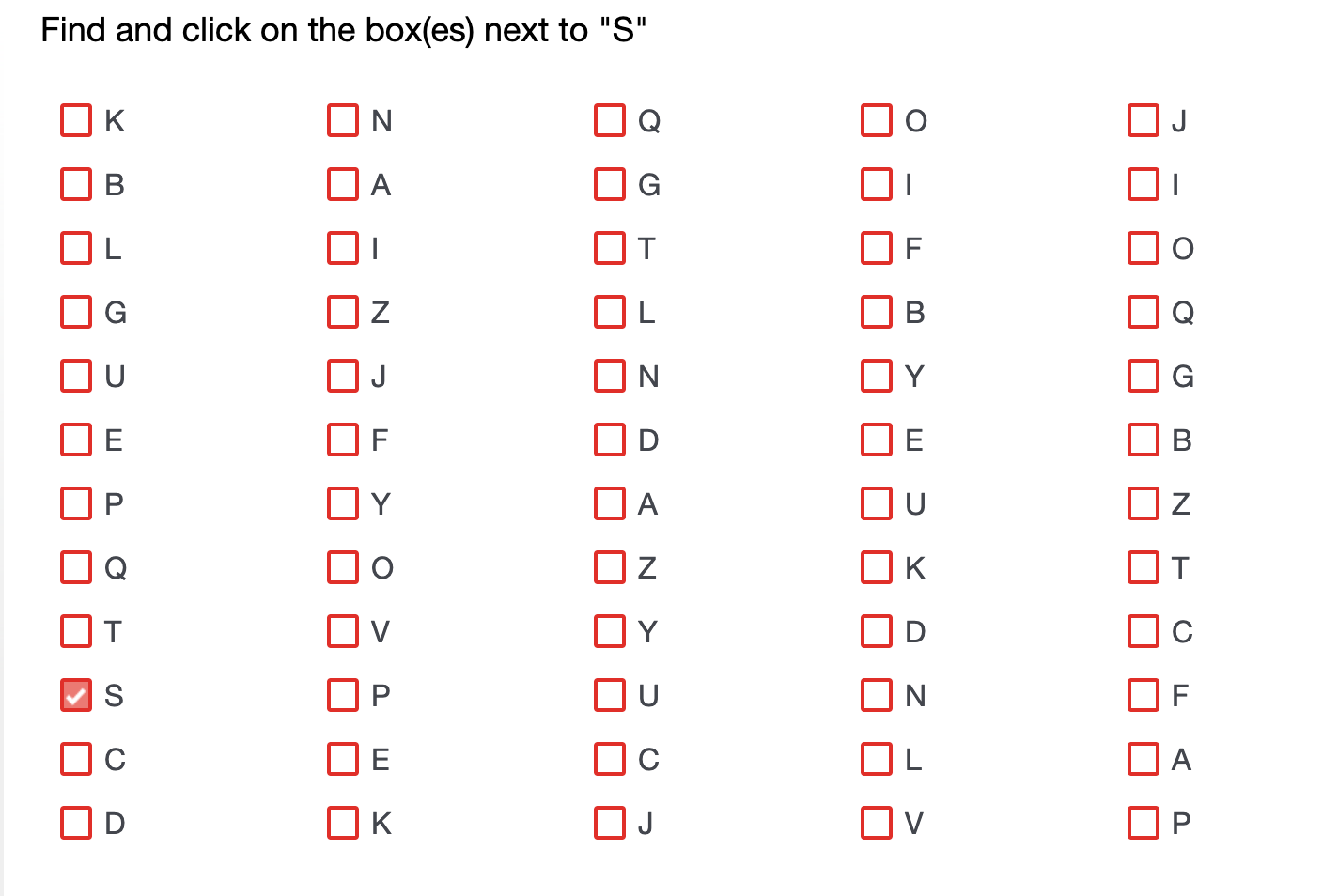
**Supplementary Figure 1.** Example of Visual Searching Task

**Supplementary Figure 2** Example of Logical Reasoning Task


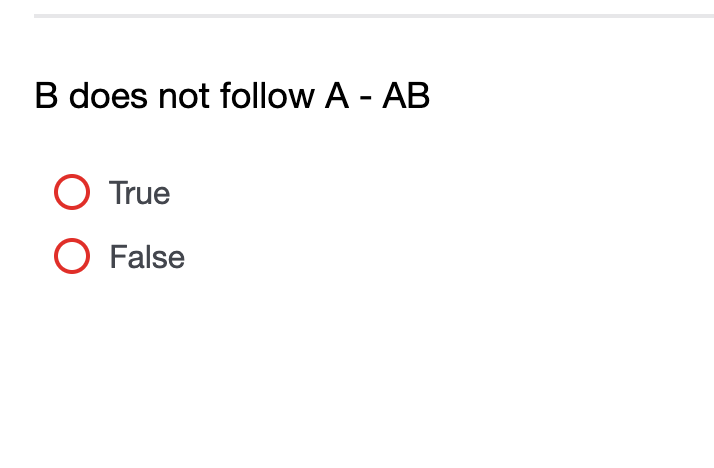

Supplement: Supplementary file 1 [file Table_1.DOCX]
